# A Rare Cause of Pancytopenia From Lepromatous Leprosy Diagnosed in Bone Marrow Aspiration: A Bolt From the Blue

**DOI:** 10.7759/cureus.69223

**Published:** 2024-09-11

**Authors:** Rugma Shine, Kalaivani Amitkumar, Shivasekar Ganapathy, Jaison J John, Jano Roy SG

**Affiliations:** 1 Department of Pathology, SRM Medical College Hospital and Research Center, SRM Institute of Science and Technology (SRMIST), Chengalpattu, IND

**Keywords:** bone marrow aspirate, drug-induced pancytopenia, fite-faraco stain, foamy histiocytes, lepromatous leprosy, leprosy in bone marrow, mycobacterium leprae, pancytopenia, virchow cells

## Abstract

Hansen’s disease is caused by *Mycobacterium leprae*. The clinical presentation of lepromatous leprosy is broad, affecting patients with reduced T-cell immune response and causing anergy. Usually, the patient presents with numerous red to brown nodules over the face and auricles and is diagnosed by skin biopsy. We hereby report an unusual case of a 40-year-old man who presented with altered sensorium and fever. Lepromatous leprosy was diagnosed initially in the bone marrow aspiration without any clinical suspicion or previous skin biopsy confirmation. Bone marrow infiltration by lepra bacilli is very rare, with only a few cases reported in the literature so far.

## Introduction

Leprosy, better termed Hansen’s disease, is a chronic infectious disease caused by a bacterial organism, *Mycobacterium leprae* [1]. It is an obligatory intracellular bacillus that is acid-fast, gram-positive, and shows tropism for Schwann cells in peripheral nerves and phagocytes in the skin [2]. Leprosy can appear in a variety of ways in clinical settings, depending on the host immune system. It typically affects the skin and peripheral nervous system, though occasionally it also affects mucous membranes [3]. Rarely can leprosy manifest as bone marrow infiltration; the literature has only a small number of case reports that describe leprosy diagnosed through bone marrow aspiration or biopsy.

## Case presentation

A 40-year-old man was brought to casualty with altered sensorium, fever with chills and rigor for six days, along with multiple episodes of vomiting and seizures. He was a known case of epilepsy since childhood and was on carbamazepine. The initial laboratory investigations revealed pancytopenia (Table 1).

**Table 1:** Laboratory parameters showing pancytopenia WBC: White blood cell; RBC: Red blood cell; PCV: Packed cell volume; HD: Hematology diagnostics; SLS-Hb: Sodium lauryl sulfate hemoglobin

Investigations	Method	Result	Range	Unit
Hemoglobin	Automated - SLS-Hb	6.3	Male: 13-17	g/dL
Total WBC count	Automated - HD focusing/flowcytometry	1,760	4,000-11,000	/cumm
Total RBC count	Automated - HD focusing/flowcytometry	2.6	Male: 4.5-5.5	million/cumm
PCV	Automated - RBC pulse height detection	22	Male: 40-50	%
Platelet count	Automated - HD focusing/flowcytometry	47,400	150,000-450,000	/cumm
Neutrophils	Flowcytometry	76.4	40-80	%
Lymphocytes	Flowcytometry	19.3	20-40	%
Eosinophils	Flowcytometry	0.0	1-4	%
Basophils	Flowcytometry	0	0-2	%
Monocytes	Flowcytometry	2.3	2-10	%
Others - Metamyelocyte	-	2	-	%
Reticulocyte count	-	0.2	-	%

**Figure 1:**
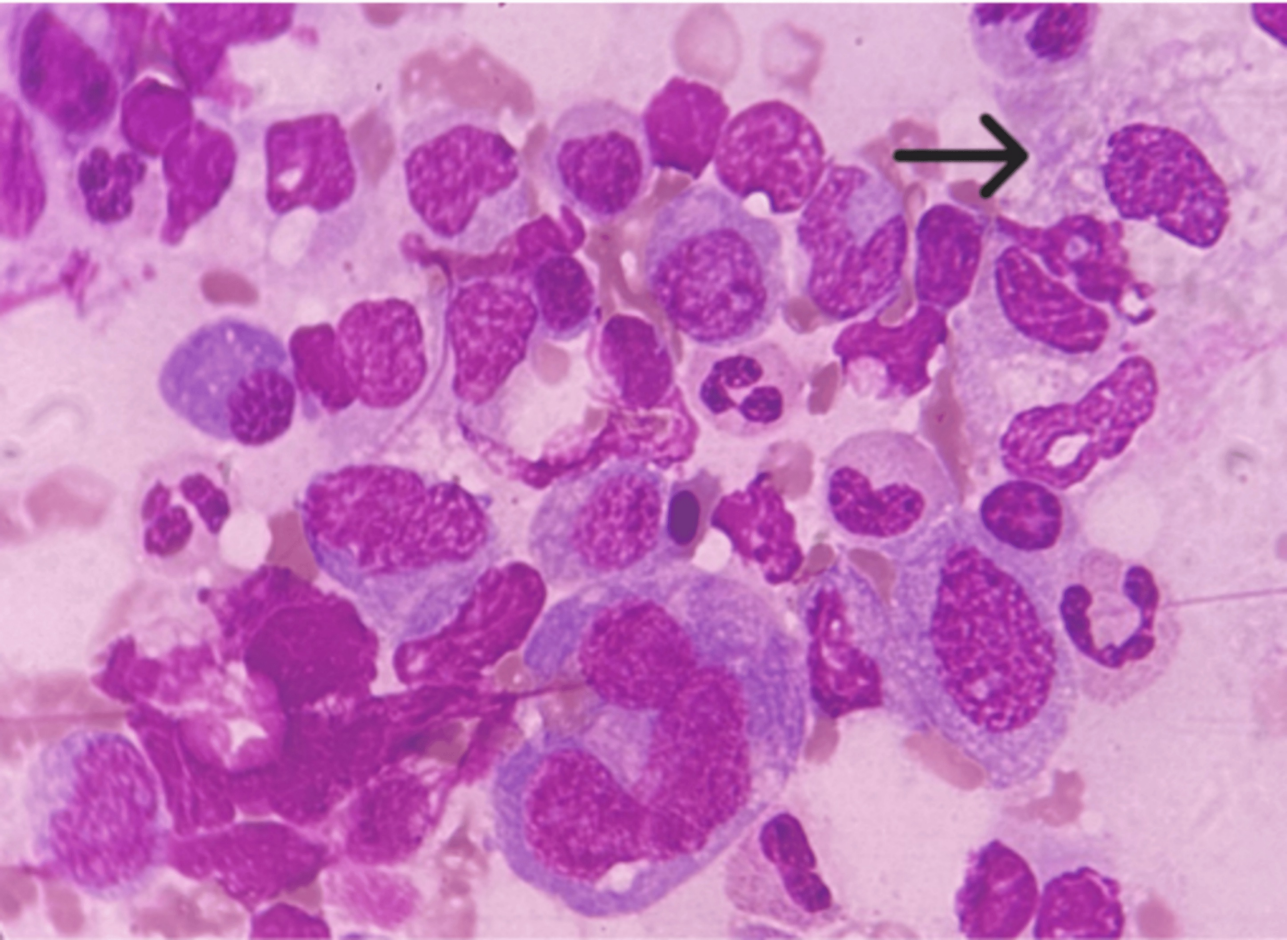
Bone marrow aspiration smears showing large histiocytes (black arrow; Leishman stain, 100x)

Bone marrow aspiration was performed, suspecting carbamazepine-induced pancytopenia, which showed suppressed erythropoiesis, increased myelopoiesis with dysmyelopoietic features, and megakaryocytosis. The reticuloendothelial activity was markedly increased, with sheets of large histiocytes (Figure 1). 

**Figure 2:**
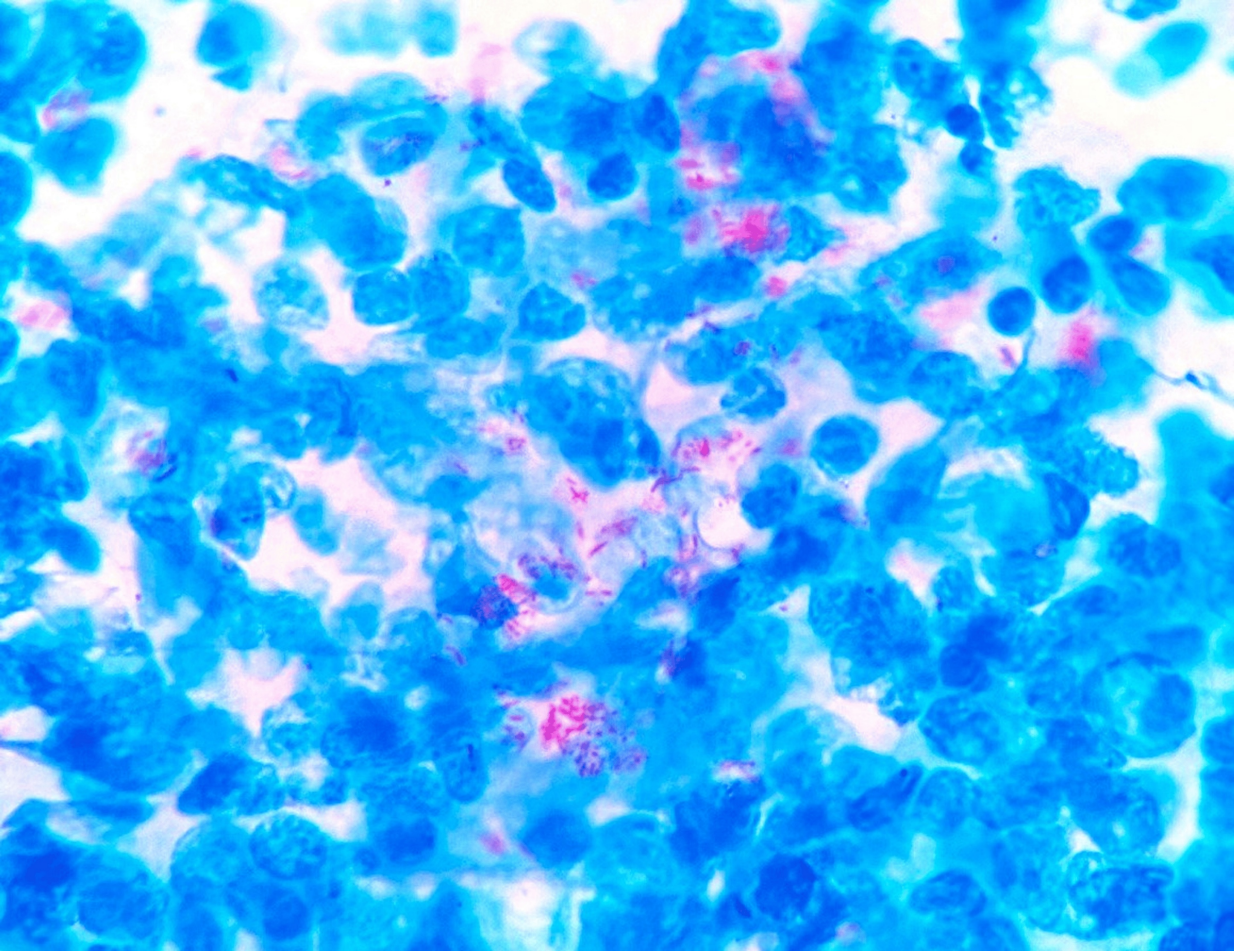
Clusters of lepra bacilli (Fite-Faraco, 100x)

With the suspicion of leprosy, a Fite-Faraco stain was performed, which showed strong positivity (4+) for lepra bacilli, consistent with lepromatous leprosy (Figure 2).

**Figure 3:**
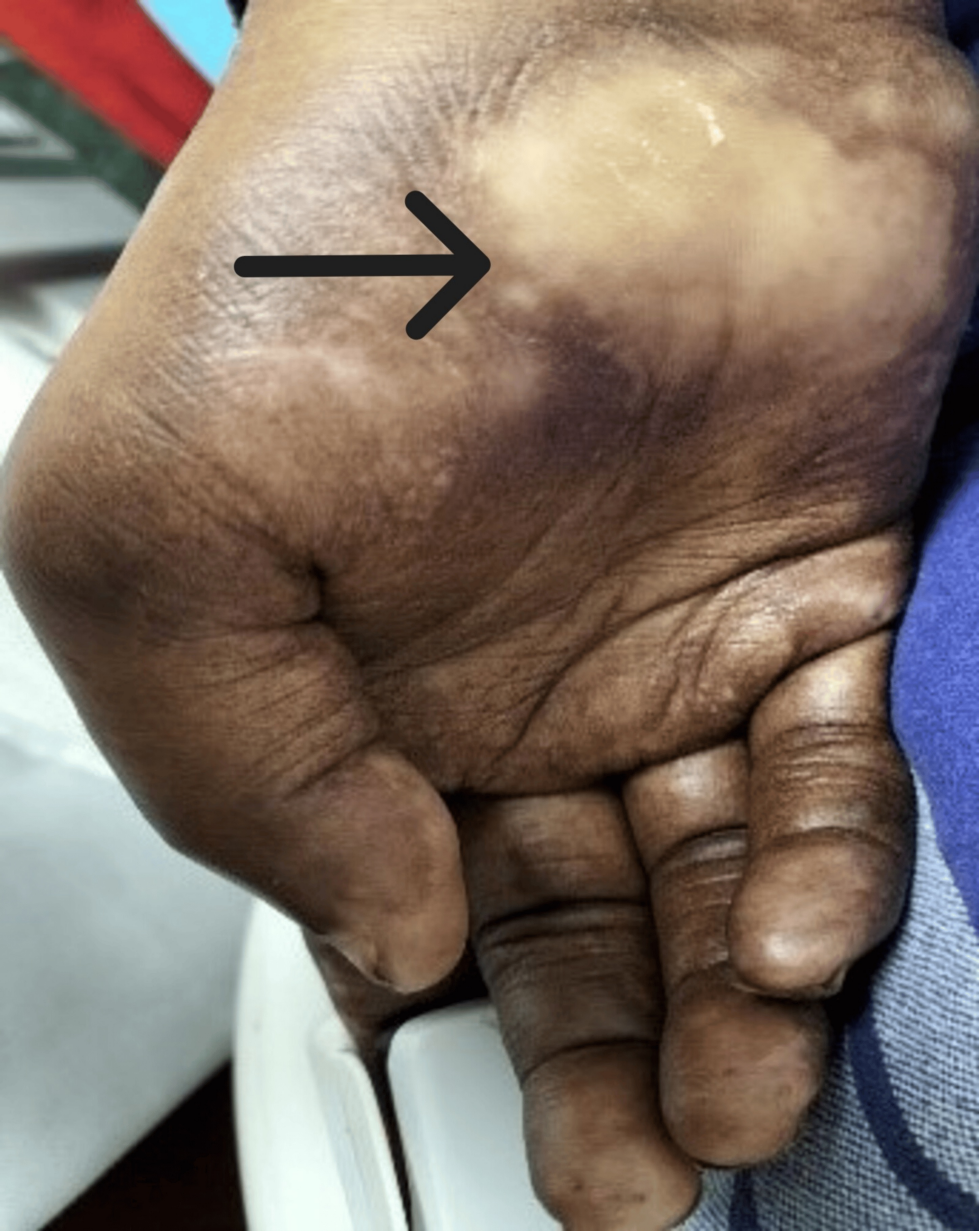
Multiple hypopigmented patches over the hand (black arrow)

**Figure 4:**
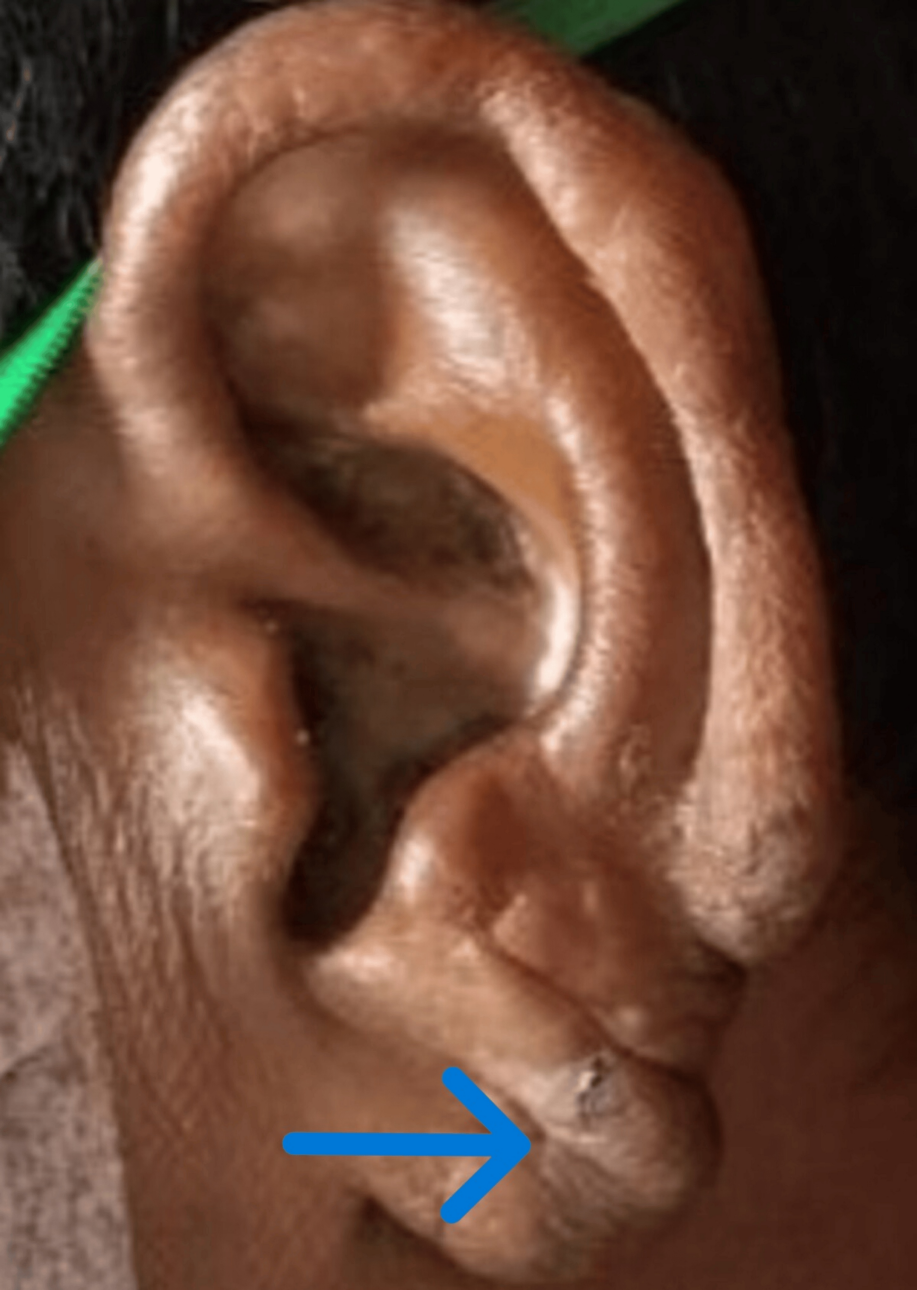
Multiple nodules over the ear lobule (blue arrow)

After the bone marrow diagnosis, a detailed clinical examination was recommended, which revealed a few hypopigmented scaly lesions and nodules over the scalp, ears, hands, bilateral knees, and foot (Figures 3-4), followed by a slit skin smear examination revealing strong positivity for lepra bacilli, suggestive of multibacillary borderline lepromatous leprosy (Table 2).

**Table 2:** Slit skin smear examination AFB: Acid-fast bacilli

Slit skin smear	Result
Right ear lobe	AFB seen (3+)
Left forehead	AFB seen (3+)
Right chin	AFB seen (3+)
Left buttock	AFB seen (2+)
Result	AFB seen

The patient was started on treatment with a supervised monthly dose of rifampin (600 mg), along with ofloxacin (400 mg), minocycline (100 mg), and an unsupervised daily dose of minocycline (100 mg) and ofloxacin (400 mg). The patient is on monthly follow-up at the outpatient clinic, with good compliance with the suggested therapy. No new skin lesions or sensory loss developed following therapy. Improvement in cytopenia and reduction in the size of existing lesions were noted after six months of initiation of treatment.

## Discussion

Leprosy is a chronic granulomatous disease, more common in equatorial as well as semitropical parts of the world, while it is present worldwide [4]. *M. leprae*, the obligatory intracellular pathogen that causes leprosy, grows slowly, with a doubling time of 10-14 days and can live for up to 45 days outside of its human host [5,6].

This bacterium is typically present in macrophages, keratinocytes, and histiocytes in infected persons, as well as in the Schwann cells of peripheral nerves, leading to demyelination, axonal malfunction, and dermatological lesions [7,8].

The World Health Organization categorizes leprosy based on symptoms, including visible lesions and bacilli in skin smears [8,9]. A "paucibacillary" infection is defined as one that results in one to five patches over the skin and no bacilli in smears taken from the skin. A person is considered "multibacillary" if they have five or more patches over the skin and the presence of bacilli in the smears taken from the skin [8]. Depending on the immune response of the individual to the infectious agent, the disease is classified as tuberculoid (TT), borderline tuberculoid (BT), borderline borderline (BB), borderline lepromatous (BL), and lepromatous (LL). Lepromatous leprosy is extremely dreadful, resulting in significant nerve damage and physical disability.

One feature noted in the cells infected with bacilli is the presence of lipid droplets, accumulation of cholesterol, oxidized phospholipids, and fatty acids that results in the formation of "foamy cells" [10].

*M. leprae* is found in nearly every bodily system in leprosy patients, but it is particularly prevalent in the reticuloendothelial system, which includes the bone marrow. The live organisms that persist in the bone marrow could be a possible explanation for the high relapse rate [11]. Bacilli persisting in the lymphovascular system deposit in the spleen, liver, lymph nodes, and bone marrow, which are the cause of the high concentration of *M. leprae* [12]. The bacterial burden in lepromatous leprosy is estimated to be 10^5 organisms/mL of blood, resulting from the inability of the immune system to fend off the infectious disease [13].

Traditionally, lepromatous leprosy involving the bone marrow is associated with the growth of foamy histiocytes, or "Virchow cells," which carry lepra bacilli [14]. When there are foamy histiocytes in the bone marrow, the differential diagnosis is quite wide. In several storage diseases, such as Gaucher disease, Niemann-Pick disease, and Wolman disease, foamy histiocytes are noteworthy. Diseases like leishmaniasis, brucellosis, listeriosis, tularemia, mycoses, and various bacterial diseases can also cause foamy histiocytes and loose granulomas [15]. Moreover, common infective etiologies of pancytopenia include leishmaniasis, disseminated tuberculosis, malaria, and enteric fever, among others [16].

In leprosy patients, fever and cytopenias are common, particularly in those receiving multidrug therapy. Fever is frequently attributed to an immunological reaction, and cytopenias are often caused by medication. *M. leprae* involvement in the bone marrow is extremely rare and rarely documented in the literature, and is frequently not suspected [14,17,18]. Pancytopenia may result from the presence of *M. leprae* as well as microgranulomas made up of plasma cells, lymphocytes, and foamy macrophages that obstruct the bone marrow's normal development.

It is important to note that aspirate smears should be stained with a modified Ziehl-Neelsen stain for an accurate diagnosis because conventional staining techniques, like this one, are frequently applied to trephine biopsy and may not always detect *M. leprae* due to chemical alterations caused by the decalcification process [19]. A thorough clinical evaluation for skin patches, nodules, and TB-PCR (*Mycobacterium tuberculosis-*polymerase chain reaction) from the node or marrow could aid in accurate diagnosis when lepra bacilli are detected in the bone marrow [20].

The increased incidence of recurrence after treatment completion may be explained by the presence of live bacilli in the marrow of *M. leprae* cases. For these patients, marrow evaluation using the Fite-Faraco stain to detect acid-fast bacilli is recommended in order to assess the involvement of the marrow, as well as the efficacy of the treatment [21].

## Conclusions

This case illustrates the importance of keeping leprosy as a differential diagnosis when a patient presents with fever and peripheral pancytopenia. Performing a Fite-Faraco stain is essential when there is the presence of aggregates of large granular histiocytes in the bone marrow, even though there is an absence of clinical suspicion or previous skin biopsy confirmation.
